# Degradation strategies for structural characterization of insoluble synthetic polymers by mass spectrometry

**DOI:** 10.1002/mas.21903

**Published:** 2024-08-02

**Authors:** Thierry N. J. Fouquet, Robert B. Cody, Laurence Charles

**Affiliations:** ^1^ Bausch + Lomb, Inc. Rochester New York USA; ^2^ JEOL USA Inc Peabody Massachusetts USA; ^3^ Aix Marseille Univ, CNRS, ICR Marseille France

**Keywords:** ASAP, controlled chemolysis, DART, DP‐APCI, synthetic polymers

## Abstract

With the advent of soft ionization techniques such as electrospray (ESI) and matrix‐assisted laser desorption/ionization (MALDI) to produce intact gas‐phase ions from nonvolatile macromolecules, mass spectrometry has become an essential technique in the field of polymeric materials. However, (co)polymers of very high molecular weight or with reticulated architectures still escape ESI or MALDI, mainly due to solubility issues. Strategies developed to tackle such an analytical challenge all rely on sample degradation to produce low‐mass species amenable to existing ionization methods. Yet, chain degradation needs to be partial and controlled to generate sufficiently large species that still contain topological or architectural information. The present article reviews the different analytical degradation strategies implemented to perform mass spectrometry of these challenging synthetic polymers, covering thermal degradation approaches in sources developed in the 2000s, off‐line sample pre‐treatments for controlled chemical degradation of polymeric substrates, and most recent achievements employing reactive ionization modes to perform chemolysis on‐line with MS.

## 1 INTRODUCTION

Since the advent of soft ionization techniques such as electrospray ionization (ESI) (Fenn et al., 1990) and matrix‐assisted laser desorption/ionization (MALDI) (Karas & Hillenkamp, 1988; Tanaka et al., 1988), production of intact ions has become the driving goal of most methods developed for the analysis of synthetic polymers by mass spectrometry (MS). One key motivation to generate molecular ions is the determination of molecular weight parameters, *M*_*n*_ = Σ*N_i_M_i_*/Σ*N_i_* and *M*_w_ = = Σ*N_i_M_i_* ^2^/Σ*N_i_M_i_*, that intrinsically relies on the abundance *N_i_* and molecular mass *M_i_* of oligomers present in the sample. Keeping polymer structure intact in the gas phase is also a requirement for end‐group analysis, using the MS mode to assess their global mass and performing MS/MS to generate fragments that can reveal the mass of individual chain termination as well as sequence information for copolymers (Wesdemiotis et al., 2011). Accordingly, the trend in modern MS of synthetic polymers has long been to push forward these two main soft techniques to achieve ionization of increasingly complex species (Wesdemiotis et al., 2024).

Yet, some (co)polymers still escape ESI or MALDI, mainly due to solubility issues. The ESI process is highly controlled by solvent properties, with abundance of ions strongly depending on the composition of the solution from which they are formed (Fenn, 1993; Kebarle & Tang, 1993). Solubility is obviously vital to ESI but use of the technique is also limited to polymers soluble in ESI‐friendly solvents. In MALDI, although solvents used to prepare samples are no longer present in the laser‐irradiated solid, they can affect its homogeneity and hence the ionization success. For synthetic polymers presenting severe solubility issues, solvent‐free procedures (Skelton et al., 2000; Trimpin et al., 2001) are advantageous alternatives to the dried‐droplet method classically used for MALDI sample preparation. Nevertheless, production of intact gas‐phase ions from very high molecular weight polymers or reticulated architectures still remains a challenge. The increasing number of complex structures conceived for most modern applications of polymeric materials also falls in these categories. Even in case of successful ionization, the large Gaussian‐like peak obtained for giant polymers does not reveal any structural information due to lack of resolution in such high *m/z* ranges. This has somehow led to a revival of strategies implemented when MS was first being used to analyze polymers, all relying on sample degradation to produce low‐mass species amenable to existing ionization methods. Degradation was typically achieved by sample pyrolysis in‐line with electron impact (EI) or chemical ionization (CI) of volatile components of the pyrolisate (Shimizu & Munson, 1979), using energetic beam process such as secondary ion mass spectrometry (SIMS) for destructive sampling of polymer surface (Hercules, 1993), or via off‐line sample pretreatments to further perform fast atom bombardment (FAB) (Williams et al., 1981). Pyrolysis induces extensive degradation of polymers and yields complex mass spectra, where fragments are most often specific of the repeating units (Hanton, 2001) but of too small size to provide additional structural details. Current added value of pyrolysis is best acknowledged in studies investigating thermal degradation pathways of polymeric materials (Lopez et al., 2017). SIMS also generates low molecular weight fingerprint mass spectra of quite limited value for structural analysis and is nowadays best valued in the field of polymeric materials for imaging and profiling purposes (Mei et al., 2022). Similarly, characteristic fragments ablated from insoluble polymer chains when using laser‐based techniques such as laser ablation electrospray ionization (LAESI) (Nemes & Vertes, 2007) are of too small size to provide structural information other than mass of repeating units, as shown for a series of synthetic fibers made of polyamide or polyaramid (van Geenen et al., 2017). Similar outputs were reported with plasma‐based methods, as reviewed by Blanksby and coworkers (Paine et al., 2014).

For degradation products to be helpful for structural characterization of synthetic polymers, chain degradation needs to be partial and controlled to generate sufficiently large species that still contain topological or architectural information, to be deciphered by advanced mass spectrometry techniques. The present article reviews the different degradation strategies that have been successfully implemented for MS analysis of polymers that still escape ESI and MALDI. This embraces new ionization techniques developed in the 2000s for MS analysis of thermal degradation products, off‐line sample pretreatments for controlled chemical degradation of polymeric substrates as well as most recent achievements employing reactive ionization modes to perform polymer chemolysis online with MS. All acronyms are defined throughout the text and also listed in Table 1 when related to instrumentation and in Table 2 when designating synthetic polymers.

**Table 1:** Acronyms related to mass spectrometry instrumentation and experiments.

ASAP	atmospheric solids analysis probe
CI	chemical ionization
CID	collision‐induced dissociation
DART	direct analysis in real time
DESI	desorption electrospray ionization
DP‐APCI	direct probe atmospheric pressure chemical ionization
DP‐APPI	direct probe atmospheric pressure photoionization
EI	electron impact
ESI	electrospray ionization
FAB	fast atom bombardment
FT‐ICR	Fourier transform ion cyclotron resonance
GC	gas chromatography
IM	ion mobility
LC	liquid chromatography
MALDI	matrix‐assisted laser desorption/ionization
MS	mass spectrometry
MS/MS	tandem mass spectrometry
SALDI	surface‐assisted laser desorption/ionization
SIMS	secondary ion mass spectrometry
TDPy	thermal desorption and pyrolysis

**Table 2:** Acronyms used for synthetic polymers.

ABS	acrylonitrile butadiene styrene
PA	polyamide
PAN	poly(acrylonitrile)
PAO	polyalphaolefin
PB	polybutadiene
PBA	poly(butylene adipate)
PBS	poly(butylene succinate)
PBT	poly(butylene terephthalate)
PC	polycarbonate
PCL	poly(caprolactone)
PDMS	poly(dimethyl siloxane)
PE	poly(ethylene)
PEEK	poly(ether ether ketone)
PEG	poly(ethylene glycol)
PEO	poly(ethylene oxide)
PET	poly(ethylene terephthalate)
PFPE	poly(perfluoropolyether)
PGA	poly(glycolic acid)
PI	polyisoprene
PIB	poly(isobutylene)
PLA	poly(lactic acid)
PMMA	poly(methylmethacrylate)
PP	poly(propylene)
PPG	poly(propylene glycol)
PPI	poly(propylene imine)
PPO	poly(propylene oxide)
PS	polystyrene
PTHF	poly(tetrahydrofuran)
PTT	poly(trimethylene terephthalate)
PUR	polyurethane
PVC	poly(vinyl chloride)
PVDF	poly(vinylidene fluoride)

## 2 CONTROLLED THERMAL DEGRADATION

This section includes studies employing three main atmospheric‐pressure ionization techniques developed in the 2000s for controlled thermal degradation of samples: Direct Analysis in Real‐Time (DART) (Cody et al., 2005), Atmospheric Solids Analysis Probe (ASAP) (McEwen et al., 2005) and Direct Probe Atmospheric Pressure Chemical Ionization (DP‐APCI) (Whitson et al., 2008). As for any new techniques or applications, performance of these three methods were first evaluated with soluble polymers before being applied to more challenging insoluble samples.

### 2.1 Direct analysis in real‐time (DART)

**Figure 1:**
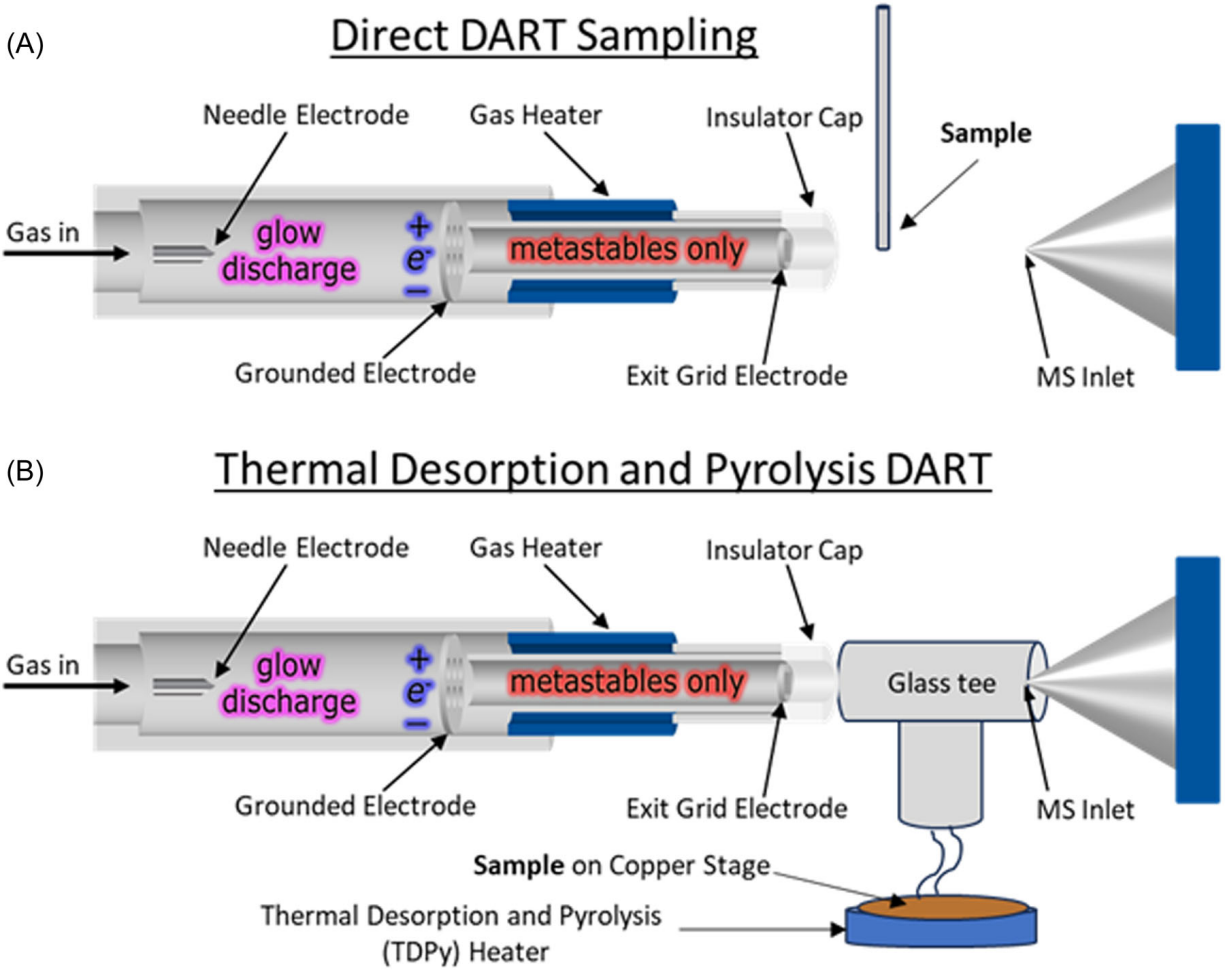
Schematic representation of a direct analysis in real‐time (DART) ion source with (A) direct sampling in the DART gas stream and (B) indirect sampling using a thermal desorption and pyrolysis device. [Color figure can be viewed at wileyonlinelibrary.com]

In DART mass spectrometry (Domin & Cody, 2014), a gas (typically helium) flows through a chamber in which a glow discharge is initiated between a needle electrode and a porous, grounded counter‐electrode (Figure 1). The flowing afterglow exiting the glow discharge chamber containing electronically excited helium atoms can be heated up to 500°C before passing through an exit electrode that is biased to favor either positive‐ion or negative‐ion formation. DART can be applied to solid‐, liquid‐, or gas‐phase samples. Solid samples exposed to the DART gas stream (Figure 1A) are volatilized and ionized, and the sample ions are carried in the gas stream into the atmospheric pressure sampling interface of the mass spectrometer. In the simplest positive‐ion DART mechanism, interactions of the excited‐state helium atoms and atmospheric moisture result in the initial formation of protonated water clusters. The protonated water clusters undergo further ion‐molecule reactions with the sample to produce protonated molecules, [M + H]^+^. Polymers can be analyzed by direct exposure to the excited‐state atoms in the hot DART gas stream, which most often results in sample pyrolysis. In an alternative approach, a solution containing the dissolved sample is deposited directly onto a target such as the sealed end of a disposable glass capillary or a porous metal mesh for transmission‐mode DART (Pérez et al., 2010).

Although this review article focuses on how DART mass spectra can provide useful structural information on the polymer itself, it should be noted that the technique is also highly valued for rapid analysis of additives present in synthetic materials since these low‐mass species are most often desorbed and ionized with high efficiency (Haunschmidt et al., 2010; Kuki et al., 2017; Rothenbacher & Schwack, 2010). DART‐MS of synthetic polymers was demonstrated since the earliest days of the technique, where production of intact protonated chains enabled the use of poly(ethylene glycol)s (PEGs) for their archetypal role of standards for external calibration of mass analyzers (Cody et al., 2005). Other polymers such as poly(dimethyl siloxane) (PDMS) were later used as standards for external calibration of DART‐MS experiments performed with Fourier transform ion cyclotron resonance (FT‐ICR), showing that the *m/z* 50–1000 normally covered by DART analysis can be usefully expanded beyond *m/z* 3000 (Gross, 2013). Then, Cody and Dane described protonated amine‐terminated polyethers as calibrants in positive mode DART‐MS whereas oligomers of poly(perfluoropropylene oxide) (Fomblin® Y), readily ionized as M^–•^, were recommended as standards in the negative ion mode (Cody & Dane, 2016).

**Figure 2:**
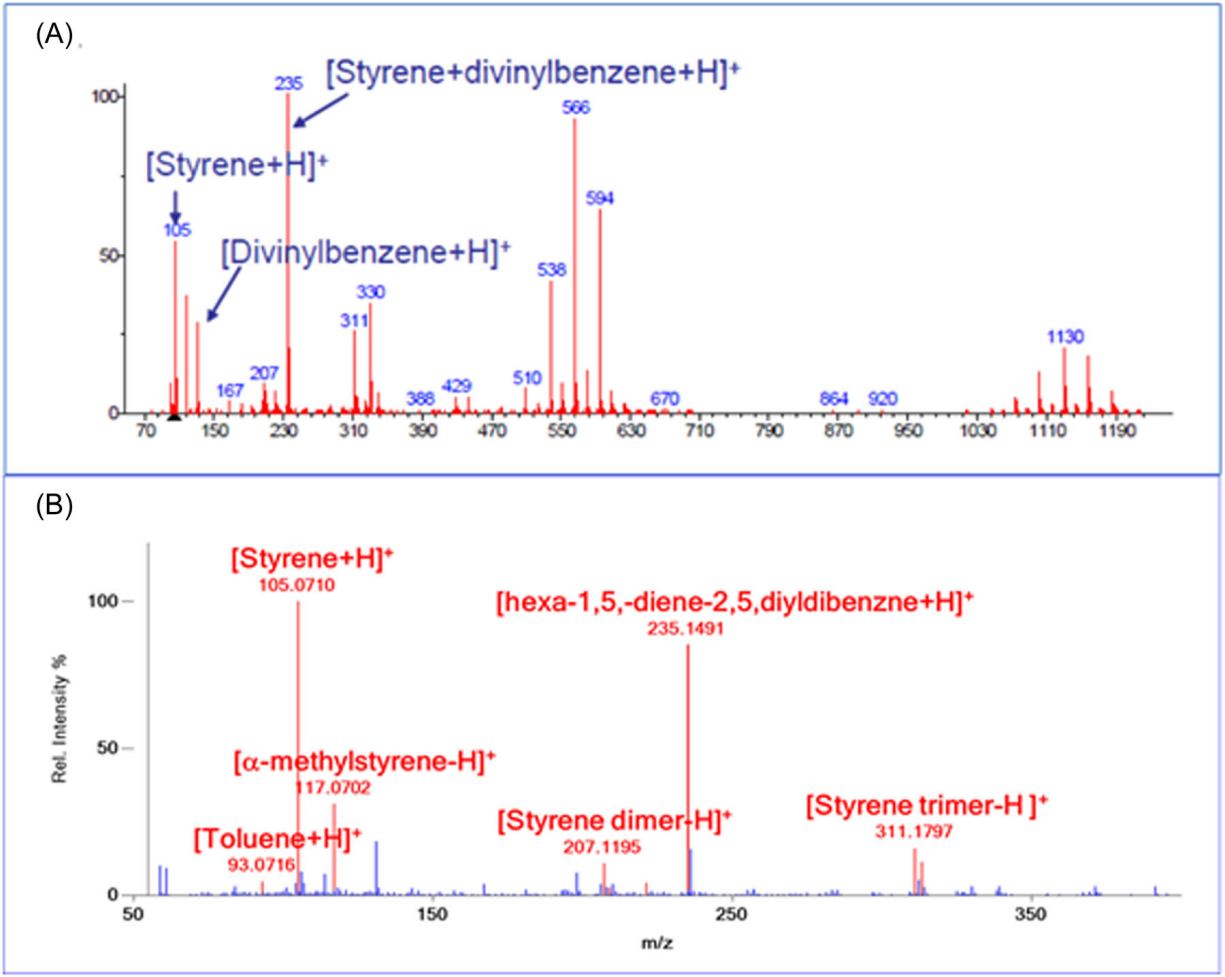
(A) Pyrolysis direct analysis in real‐time (DART) mass spectrum of polystyrene (JEOL USA, Inc, 2005). (B) DART mass spectrum of polystyrene with tentative peak assignments corresponding to known pyrolysis products from the gas chromatography–mass spectrometry literature. [Color figure can be viewed at wileyonlinelibrary.com]

Direct analysis of synthetic samples such as polymers, glues, adhesives, and resins by DART also first appeared in 2005 in a set of application notes that are still available on the JEOL website (JEOL USA, Inc, 2005). DART mass spectra of poly(lauryllactam) also known as Nylon 12, polystyrene (PS) and cellulose (cotton) showed characteristic pyrolysis fragments. Protonated n‐mers (with *n* = 1–4) were observed for poly(lauryllactam) whereas the cellulose mass spectrum showed protonated and ammoniated mono‐ and di‐saccharides with multiple water losses. The DART mass spectrum of PS shown in Figure 2A was labeled with tentative assignments based on accurate mass measurements. These were based on a poor understanding of pyrolysis and DART ionization mechanisms. With our current understanding of DART ionization of alkyl‐substituted aromatics, it is more likely that some of these peaks correspond to [M – H]^+^ for pyrolysis fragments (Figure 2B) that are well‐documented in the pyrolysis gas chromatography–mass spectrometry (GC‐MS) literature (Tsuge et al., 2011).

Capabilities of the technique in terms of accessible mass range and type of polymeric species were evaluated by Bridoux et al. who examined a series of polymers with a DART ion source coupled to an Orbitrap mass spectrometer (Bridoux & Machuron‐Mandard, 2013). Samples dissolved in tetrahydrofuran were applied to the stainless‐steel mesh screen sampling device for the DART linear rail and analyzed by transmission‐DART. At DART gas heater temperatures between 400°C and 500°C, protonated oligomers and ammonium adducts were observed for all polymers except perfluoropropyl ethers, which were detected as M^–•^ in the negative ion mode. Molecular weight and polydispersity metrics obtained from these DART‐MS data compared favorably with gel permeation chromatography analysis for polymers having a molecular weight range between 200 and 4000 Da. As supported by MS/MS experiments, intact oligomers still holding their original terminations were always detected as the major species for all tested polymers. No thermal fragmentation was observed for polyethers such as PEG‐600, poly(propylene glycol) (PPG‐1000), and poly(perfluoropolyether)s (PFPEs) with different end‐groups, or aliphatic long‐chain polyamine biopolymers (polypropyleneimines and N‐methylated polypropyleneimines). Beside major ammonium adducts detected for OH‐terminated PDMS, low abundance signals corresponding to water loss were observed: the authors suggested that these were sample impurities, rather than thermal degradation products, because the same species were also detected when lowering the DART temperature. In contrast, minor ionic species assigned to [(M–CH_2_) + H]^+^, [(M–CH_4_O) + H]^+^, and [(M–C_2_H_4_O_2_) + H]^+^ in DART mass spectra recorded for poly(methylmethacrylate) (PMMA‐1000) showed that thermal degradation occurred when analyzing this sample at 500°C. These results are consistent with data recently reported by Aouak et al. who monitored thermal decomposition of PMMA by DART‐MS: the depolymerization process starts at about 250°C, as revealed by an increased signal of protonated MMA monomers in the mass spectrum, not far from the temperature measured at the beginning of the polymer decomposition in thermogravimetric analysis (Al Khulaifi et al., 2023).

Additional research from Bridoux et al. reported on DART‐MS of defused military weapons (Bridoux et al., 2016). Micro‐Raman spectroscopy was used to first localize the explosive charge on the impaction plate which was then wiped with cotton swabs further subjected to DART(+)‐MS: in addition to explosives and plasticizers, mass data revealed a very complex distribution of polymeric binders, mainly PEG and PPG, detected either as protonated molecules or ammonium adducts. Kendrick mass defect analysis was implemented to tackle the complexity of DART‐MS data obtained for post‐blast residues and revealed the presence of poly(isobutylene) (PIB), poly(butadiene) (PB), and PS in some of the samples (Gaiffe et al., 2019). Similar forensic investigation was performed by Cizdziel et al. who readily obtained the spectral signature (i.e., ions at *m/z* 105.1, *m/z* 211.1, and *m/z* 262.2) of acrylonitrile butadiene styrene (ABS) used in the gun barrel of 3D‐printed firearms when subjecting cartridge and bullet scraping to DART‐MS analysis (Black et al., 2017).

**Figure 3:**
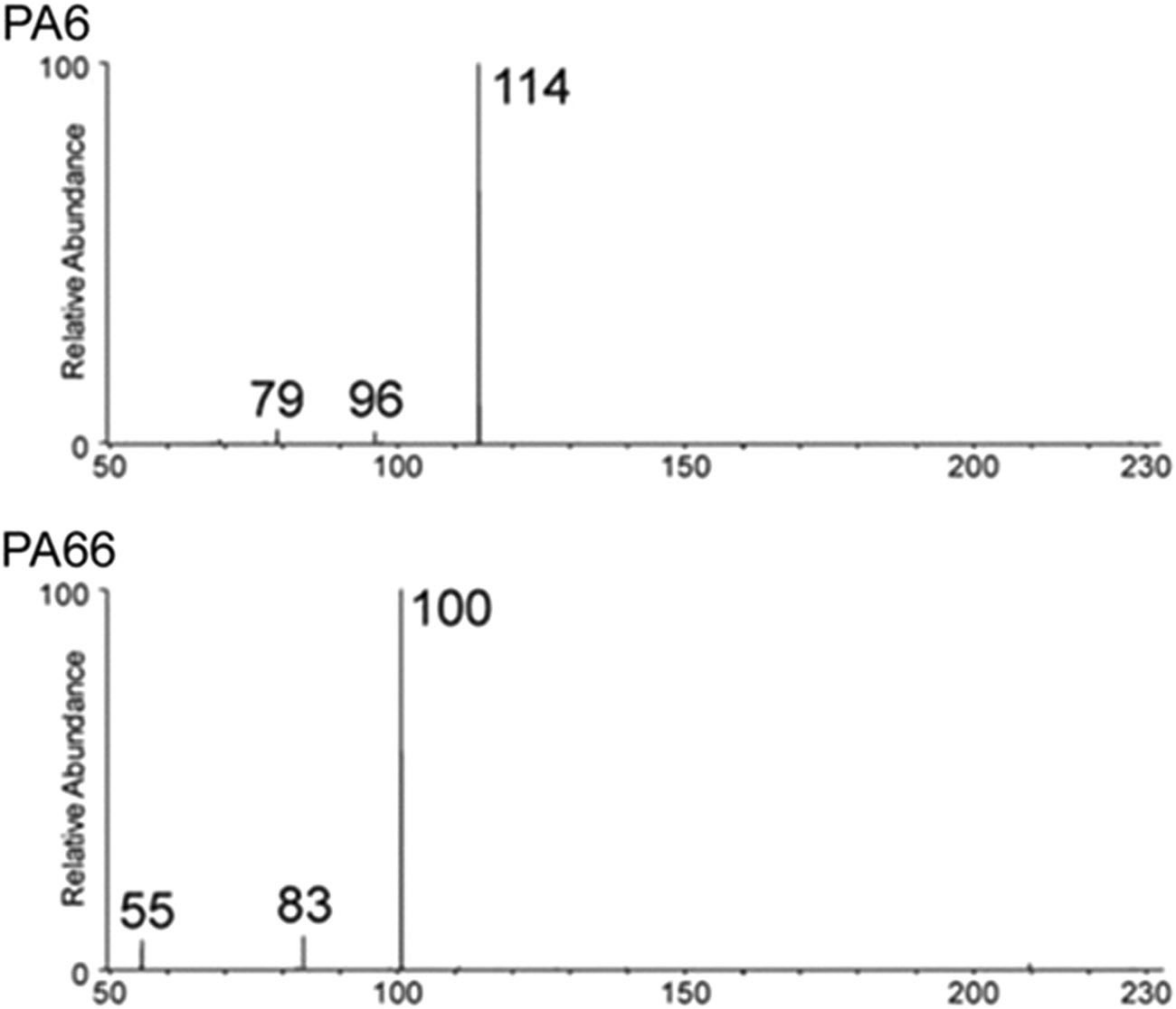
Different fragments observed in MS/MS of the *m*/*z* 227 cyclic dimers formed upon direct analysis in real‐time of PA6 (top) and PA66 (bottom) enable clear distinction of the two polymers. Adapted with permission from Abe et al. (2020). Copyright 2020 Wiley.

Nagy et al. investigated DART analysis of low‐molecular‐weight PIBs (Nagy et al., 2015). With added NH_4_Cl, chloride adducts [M + Cl]^–^ were detected in the negative ion mode for chlorine telechic and isobutylene telechic PIBs whereas deprotonated oligomers [M – H]^–^ were observed for poly(isobutylene) succinic acid. In the positive mode, DART generated ammonium adducts for all PIB samples. MS/MS analysis of these [M + NH_4_]^+^ ions provided structural information, and a model based on the Clausius‐Clapeyron equation was applied to determine the average molecular weight. Cody and Fouquet reported positive‐ion DART‐MS spectra of amine‐terminated polyethers (“Jeffamines”), PDMS, poly(lactic acid) (PLA), PMMA, and PIB‐based additives in gasoline (Cody & Fouquet, 2018). The DART mass spectra were used to demonstrate a “reverse Kendrick mass defect” method in which mass defect plots of homopolymers were rotated to zero slope to determine the monomer composition. Ackerman et al. at the US Food and Drug Administration applied DART for rapid identification of polyamides (PA, also named nylons) (Abe et al., 2020). The number of oligomer ions as well as their abundance were observed to increase with increasing DART temperature in the tested 100–500°C range. Unique diagnostic ions were found to enable MS distinction of eight different PAs whereas MS/MS was requested to distinguish PA6 from PA66 based on different dissociation reactions experienced by their *m*/*z* 227 cyclic dimer (Figure 3). Similar findings were reported that same year by Zughaibi and Steiner (2020). Pacholski et al. used DART coupled with a Fourier transform mass spectrometer to differentiate four poly(vinylidene fluoride) (PVDF) polymers based on their end‐group composition revealed by Kendrick mass defect analysis and further probed by MS/MS (Pacholski et al., 2023).

DART‐MS is also very convenient for rapid fingerprinting of environmental micro‐ and nano‐plastics (MNPs), as reported by Jobst and coworkers who used multivariate statistics to best highlight differences among plastic types and source materials (Zhang et al., 2020). Using fuzzy c‐means clustering for rapid screening of MNPs, Forbes et al. showed that DART‐MS is well suited for environmental samples that are likely to contain multiple polymers such as PS, poly(propylene) (PP), poly(ethylene terephthalate) (PET), PMMA, and nylon 6 (Forbes et al., 2023). An original application of DART‐MS was reported by AlShehri et al. to determine the miscibility of binary polymer blends (AlShehri et al., 2022). Five binary polymer blends were analyzed by DART‐MS and the results were compared with differential scanning calorimetry (DSC) and thermogravimetic analysis (TGA). Thermal fragmentation of miscible polymers showed some fragment ions containing components of both polymers, whereas immiscible polymers showed only fragment ions of the pure components.

**Figure 4:**
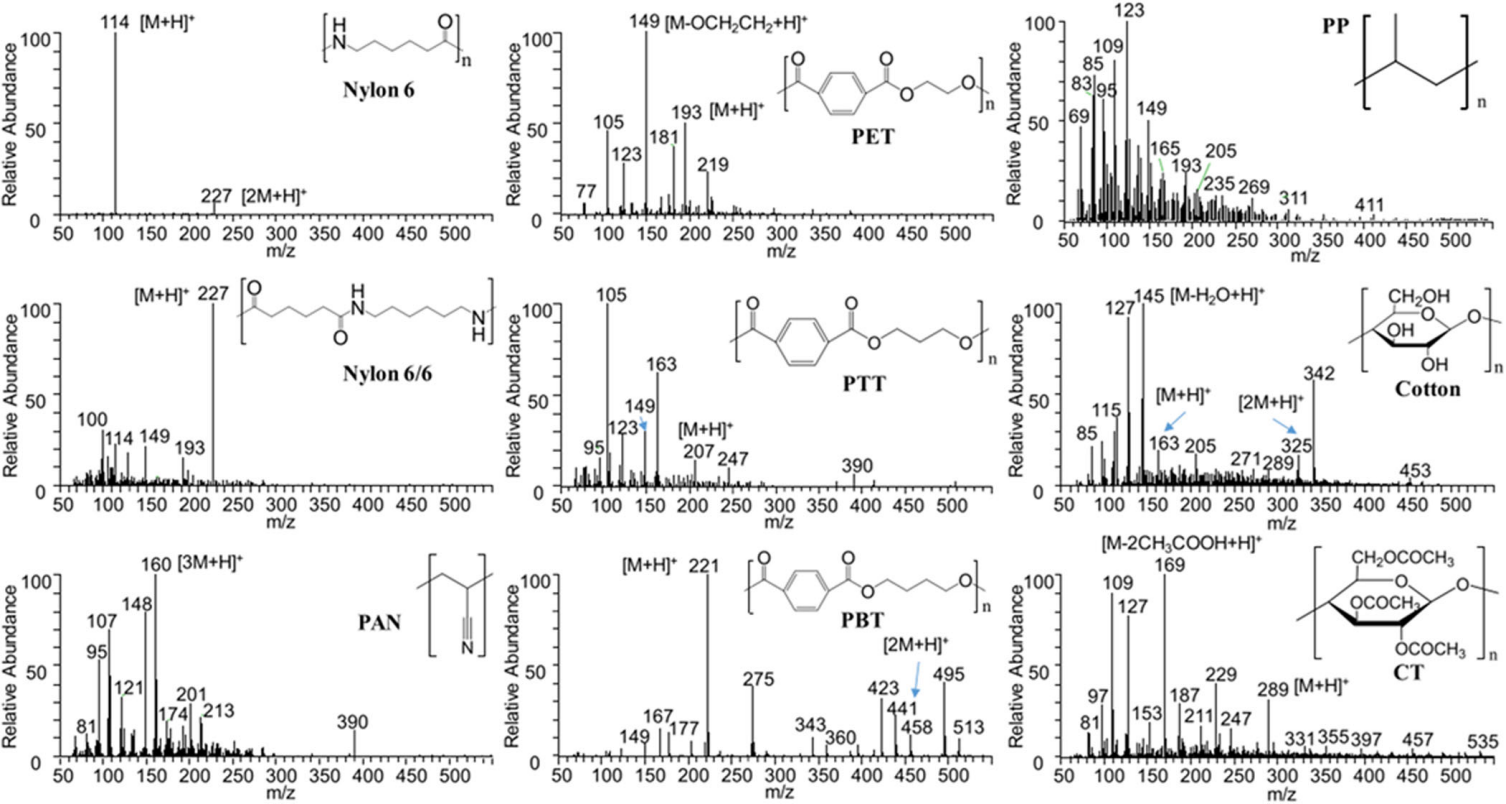
Characteristic TDPy‐DART‐MS patterns of polymeric fibers in textile materials. CT, cellulose triacetate; PAN, poly(acrylonitrile); PBT, poly(butylene terephthalate); PET, poly(ethylene terephthalate); PP, polypropylene; PTT, poly(trimethylene terephthalate). Reproduced with permission from Liang et al. (2020). Copyright 2020 American Chemical Society.

An alternative to placing the sample directly into the DART gas stream is to use a thermal desorption and pyrolysis (TDPy) device to heat the sample (Figure 1B). Evolved gases, including vapor from additives, contaminants, and thermally decomposed polymers, are directed into the DART gas stream where the vapor is ionized and transported into the mass spectrometer atmospheric sampling interface (Cody et al., 2020). TDPy DART provides a temperature‐regulated heating program, analogous to thermal desorption/pyrolysis GC‐MS. The thermal desorption profiles show the temperature‐dependent desorption of additives and contaminants and polymer pyrolysis fragments. TDPy DART is particularly beneficial for the analysis of polyolefins where the ionization of more polar additives can suppress the DART ionization of relatively nonpolar polyolefins. TDPy DART is also convenient for the analysis of small particles, fibers, and powders because the samples are not exposed directly to the vigorous flow of the DART gas stream. For example, Liang et al. applied TDPy DART to the forensic identification of fibers (Liang et al., 2020). In this study, chemometric methods including principal component analysis (PCA) and Pearson product‐moment correlation (PPMC) were applied to the classification of 40 commercial fiber samples (Figure 4), with successful identification of even blended polymers.

TDPy DART‐MS is also valued in studies investigating thermal decomposition of synthetic polymers. Sato et al. studied the temperature dependence for desorption of intact oligomers and thermal decomposition products of PS (Sato, Nakamura, Takei, et al., 2020). The initial appearance of intact oligomers at 200°C was followed in succession by the appearance of pyrolysis products still containing the butyl end‐group (270°C) and pyrolysis products from internal chain fragmentation (300°C) resulting from oxidative cleavage. Interestingly, the ratio of the fragments containing the butyl end‐group to the internal fragments was found to be inversely proportional to the number average molecular weight, suggesting the possibility of PS molecular weight determination by DART. Thermal degradation of perfluorosulfonic acid (Nafion™) ionomers, that have applications in proton exchange membrane fuel cells, was also investigated by TDPy DART‐MS(/MS) (Yamaguchi, 2021). Thermal decomposition begins at 350°C with side chain detachment, followed by main chain scission at 400°C and further decomposition above 500°C. Yamane et al. demonstrated that the rapid heating process employed in TDPy DART‐MS experiments enabled determination of the block sequence of linear triblock copolyethers with no prior knowledge of the synthesis conditions, since the repeated structure of the outer blocks was emphasized in mass spectra recorded in the early analysis stage (Yamane et al., 2021). Finally, an interesting variation on TDPy DART‐MS is the combination of hot‐stage microscopy with DART‐MS reported by Harding and co‐workers (Ashton et al., 2017; Ashton et al., 2021). Optical, thermal, and mass spectrometric information were combined to examine the thermal expansion and release of volatiles from surgical‐grade PDMS and to identify poly(ethylene) (PE) and PS microplastics in beach sand.

### 2.2 Atmospheric pressure solids analysis probe (ASAP)

**Figure 5:**
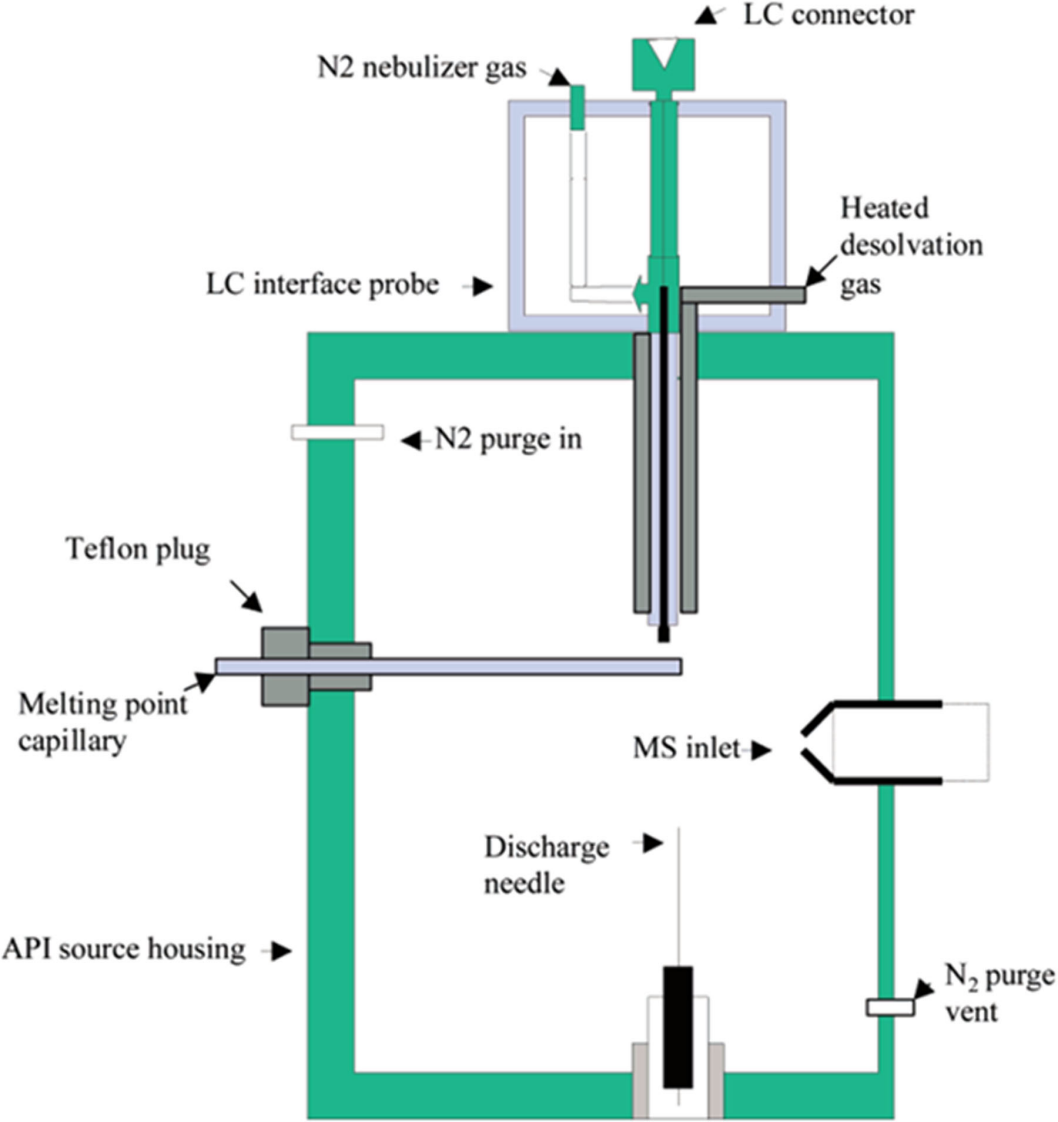
Schematic representation of the atmospheric pressure solids analysis probe source. Reproduced with permission from McEwen et al. (2005). Copyright 2005 American Chemical Society. [Color figure can be viewed at wileyonlinelibrary.com]

ASAP uses a solid probe to insert a sample into an atmospheric pressure chemical ionization source (McEwen et al., 2005), the sample being vaporized in the hot nitrogen gas stream and ionized by the corona discharge (Figure 5). Solid samples are commonly deposited onto disposable glass melting point tubes for analysis by ASAP. As with DART, ASAP‐MS also reveals additives present in polymeric materials, as reported by Du et al. who created a searchable library permitting the identification of additives in PP and PMMA samples (Du et al., 2014).

**Figure 6:**
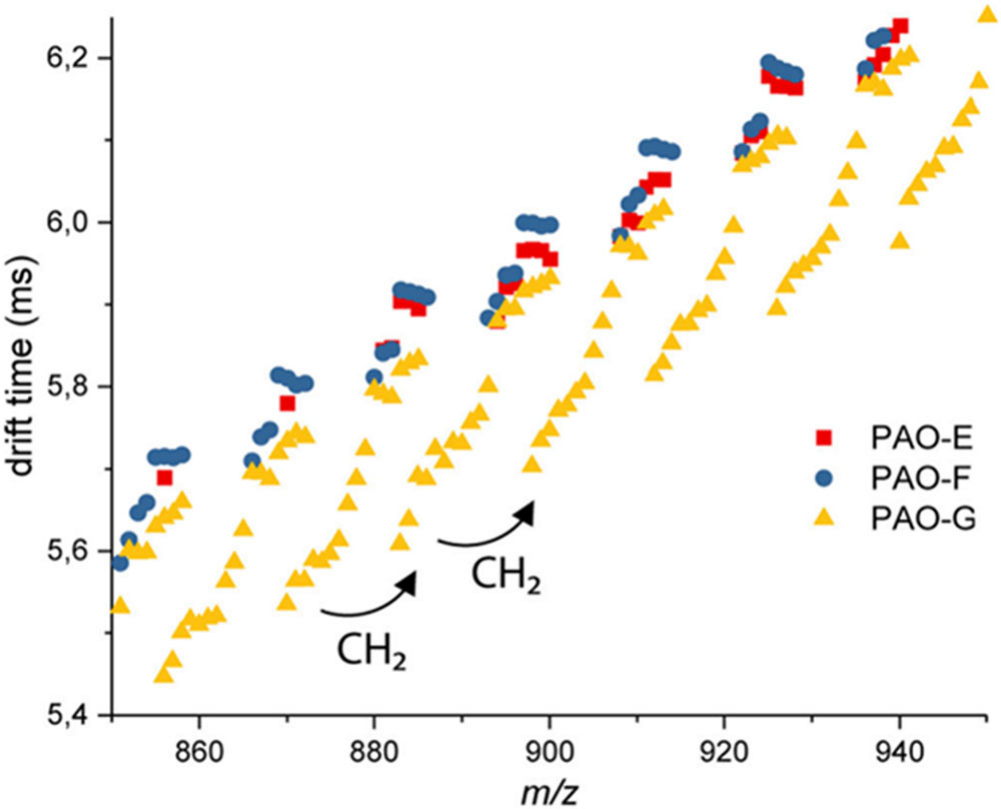
2D IM‐MS plot obtained for atmospheric pressure solids analysis probe pyrolysis products of polyalphaolefins (PAOs) of different grades. Reproduced with permission from Siqueira et al. (2018). Copyright 2018 American Chemical Society. [Color figure can be viewed at wileyonlinelibrary.com]

The first publication on ASAP included a mass spectrum of PEG‐440 used as a mass reference standard (McEwen et al., 2005). While PEG oligomers were detected as protonated species, ASAP of PS chains (*M*_*n*_ 1770 Da) was observed to generate radical cations, which facilitates directional MS/MS sequencing and end‐group analysis (Smith et al., 2012). Similarly, when detected as M^+•^, pyrolysis products generated during ASAP of low‐solubility poly(ether ether ketone) (PEEK) had their MS/MS more structurally informative than dissociation of the protonated molecules (Cossoul et al., 2015). The complexity of ASAP mass spectra highly depends on the type of investigated samples. ASAP of some polymers produces small cyclic oligomers only, yielding the same very simple mass spectra regardless of the initial size of chains. This was reported for PET containers or Nylon‐6 fibers (Trimpin et al., 2009), as well as for H,OH‐ended PDMS with *M*_w_ up to 110,000 Da (Fouquet, Barrère‐Mangote, et al., 2015). Protonated cyclic n‐mers were also observed for biodegradable polyesters such as PLA (with *M*_w_ of 10 kDa or 76 kDa) or poly(butylene succinate) (PBS) (Barrère et al., 2014). Most often, however, ASAP of synthetic polymers produces complex mass spectra, which explains why numerous studies involve separation of ionized species by ion mobility (IM) before their MS analysis. ASAP‐IM‐MS coupling was first reported for PP (Barrère et al., 2012), where the five main product distributions were found to be the same as obtained upon PP pyrolysis (Lattimer, 1993). Interestingly, this study also reports different pyrolysis products for PP sample that did not contain any antioxidant, suggesting that ASAP could usefully be employed to get insight into stabilizer activity. Comparison of ASAP mass data recorded for isotactic PP, atactic PP, and PE revealed the same oxidized pyrolysis products but in different relative ratios (Farenc et al., 2017). With series of pyrolysis products evidenced in IM‐MS 2D plots, ASAP of cyclic olefin copolymers grafted with aryl layers from aryldiazonium salts was found to provide complementary information to atomic force microscopy (AFM) and infrared spectroscopy data recorded for the same samples (Vieillard et al., 2015). Polyalphaolefins (PAOs) examined by ASAP coupled with IM‐MS showed differences in the mass spectra measured with different gas temperatures and cone voltages (Siqueira et al., 2018). Drift time versus *m/*z plots permitted to highlight different distributions of pyrolysis fragments depending on the PAO grades (Figure 6) and to distinguish compact and linear PAO structures. Differences in PAOs containing different number of isomeric subunits were also apparent in the width of the ion mobility peaks.

**Figure 7:**
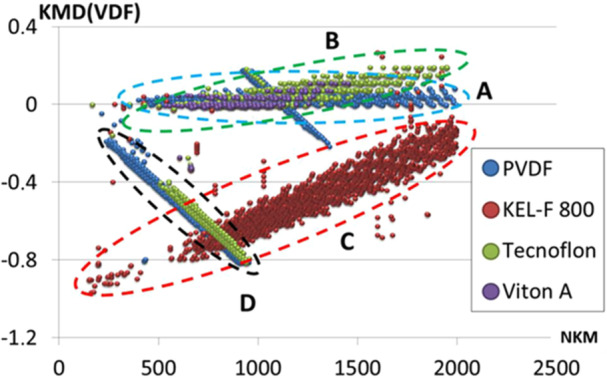
Use of vinylidene fluoride (VDF, C_2_H_2_F_2_) as the base unit in Kendrick mass defect analysis of ASAP‐MS data to distinguish different fluorinated polymers. Reproduced with permission from Gaiffe et al. (2018). Copyright 2018 American Chemical Society. [Color figure can be viewed at wileyonlinelibrary.com]

Another mean to tackle the complexity of ASAP mass spectra is the use of ultrahigh resolution mass analyzer combined with Kendrick mass defect (KMD) data analysis. This approach was implemented to establish the ASAP‐MS fingerprint of insoluble fluorinated polymers (Gaiffe et al., 2018). As depicted in Figure 7, KMD analysis using vinylidene fluoride (VDF, C_2_H_2_F_2_) as the base unit enabled clear distinction of ASAP products detected for PVDF from those recorded for copolymers composed of VDF units and either chlorotrifluoroethylene units (KEL‐F 800) or hexafluoropropylene units (Viton A and Tecnoflon).

**Figure 8:**
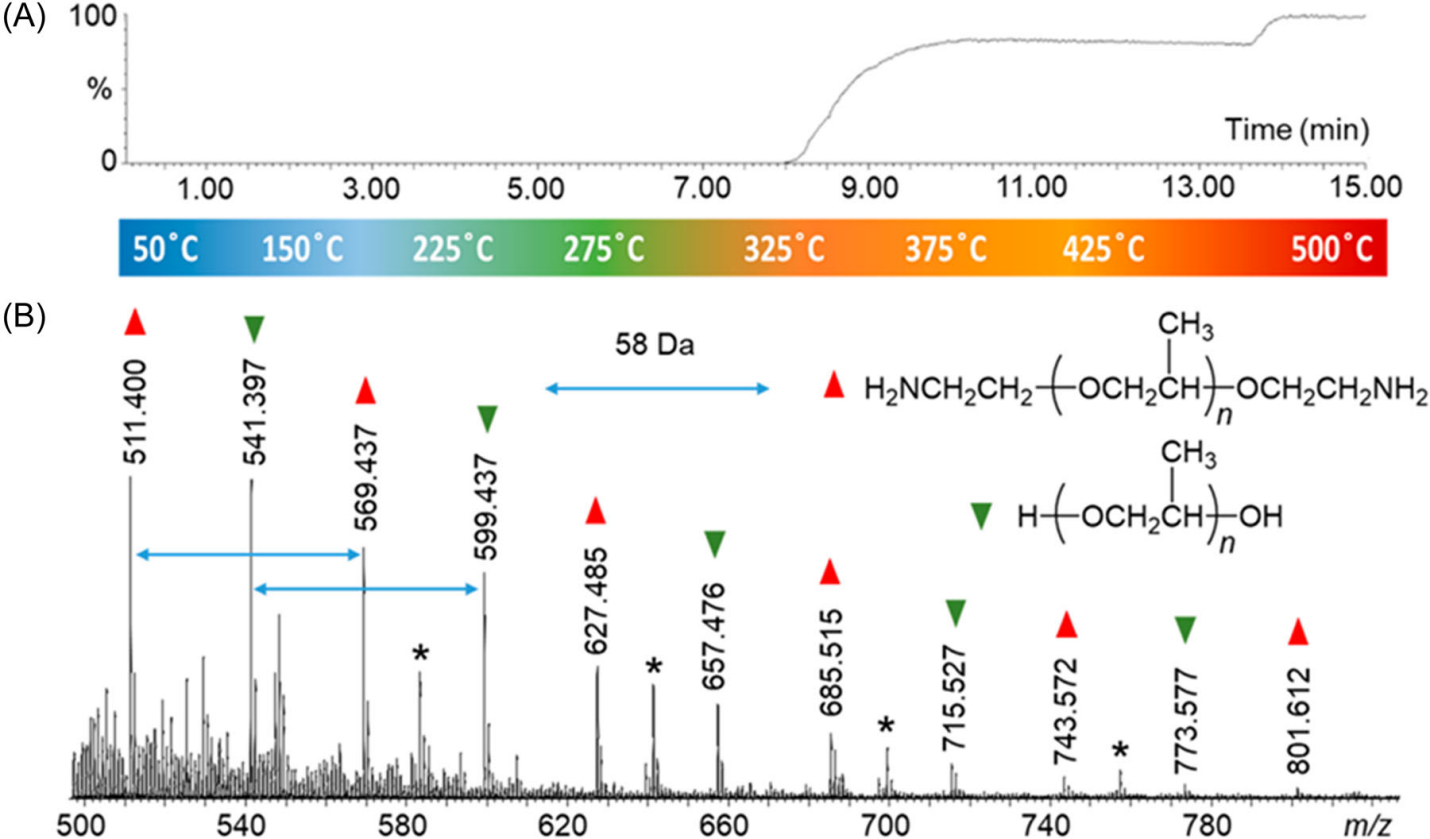
(A) ASAP‐MS thermogram from 50°C to 500°C of an unknown engine deposit. (B) Extracted mass spectrum from 8.00 to 8.50 min showing two PPG distributions (annotated by triangles) as well as one series of poly(isobutylene) (PIB) oligomers (designated by asterisks). Reproduced with permission from Snyder and Wesdemiotis (2021). Copyright 2021 American Chemical Society. [Color figure can be viewed at wileyonlinelibrary.com]

Lebeau and Ferry characterized polyurethanes (PURs) with ASAP by increasing the nitrogen desolvating gas temperature over a range from 350°C to 600°C (Lebeau & Ferry, 2015). After detection of additives at the lowest gas temperatures, peaks associated with methylene diisocyanate (MDI) were observed as the gas temperature increased, and characteristic pyrolysis fragments were detected for polyether and polyester PURs at the highest temperatures. Similar findings were reported for different thermoplastic PURs by Alawani et al. who also examined styrene‐butadiene elastomers by ASAP (Alawani et al., 2023). Capitalizing on the resolving capabilities offered by the IM‐MS coupling, large degradation products (>*m/z* 500) generated when increasing the ASAP gas temperature to 450°C could be clearly observed and assigned to chains composed of styrene and butadiene comonomers and holding different end‐groups. Using thermometric gradient ramping, the added value of ASAP was also demonstrated by the Wesdemiotis' group while investigating the nature of insoluble particles found in vehicular engine deposits (Snyder & Wesdemiotis, 2021). Different types of polymeric additives were detected as a function of their boiling points, as exemplified in Figure 8 with protonated poly(propylene oxide) (PPO) oligomers with different end‐groups as well as PIB species detected when the ASAP gas temperature was raised from 325°C to 337°C. These data were consistent with pyrolysis residues obtained for standards of polymeric lubricants directly subjected to ASAP (Barrère et al., 2014) or after separation by high‐performance thin‐layer chromatography (Beaumesnil et al., 2020). Isothermal heating steps were implemented to characterize single microplastic particles with ASAP (Vitali et al., 2023). Samples were incubated with Nile Red dye to facilitate identification of microplastic particles by fluorescence microscopy. The ASAP gas temperature was first set to 375°C to remove surface contamination. After a 1‐min hold at 375°C, the gas temperature was increased to 600°C for pyrolysis and microplastic particles were characterized based on the specific mass spectra obtained for a wide range of polymeric components. ASAP was also successfully employed to characterize small (*M*_*n*_ 875 Da) but poorly soluble amphiphilic PEG‐*b*‐PE block copolymers end‐capped by an azidoethyl glycoside group (Eissa et al., 2013). Using a nitrogen gas temperature of 400‐450°C, the ASAP mass spectrum displayed signals for the PE block, the azidoethyl glycoside end‐group as well as the protected sugar moiety, these three main features enabling full description of the scrutinized samples. Finally, combining ASAP with IM‐MS(/MS) revealed unique information about the crosslinking chemistry and the identity of precursor materials used to prepare covalently crosslinked hydrogels (Endres et al., 2021). In the ASAP source maintained at 450°C, these networks of a virtually infinite molecular weight were broken down to short PEG oligomers, best ionized as protonated molecules by placing a vial of methanol in the source. Prior knowledge of the decomposition reactions at work during degradation of PEG was essential for ASAP‐MS data interpretation, further supported by MS/MS end‐group analysis of PEG pyrolyzates (Endres, 2019).

### 2.3 DP‐APCI

**Figure 9:**
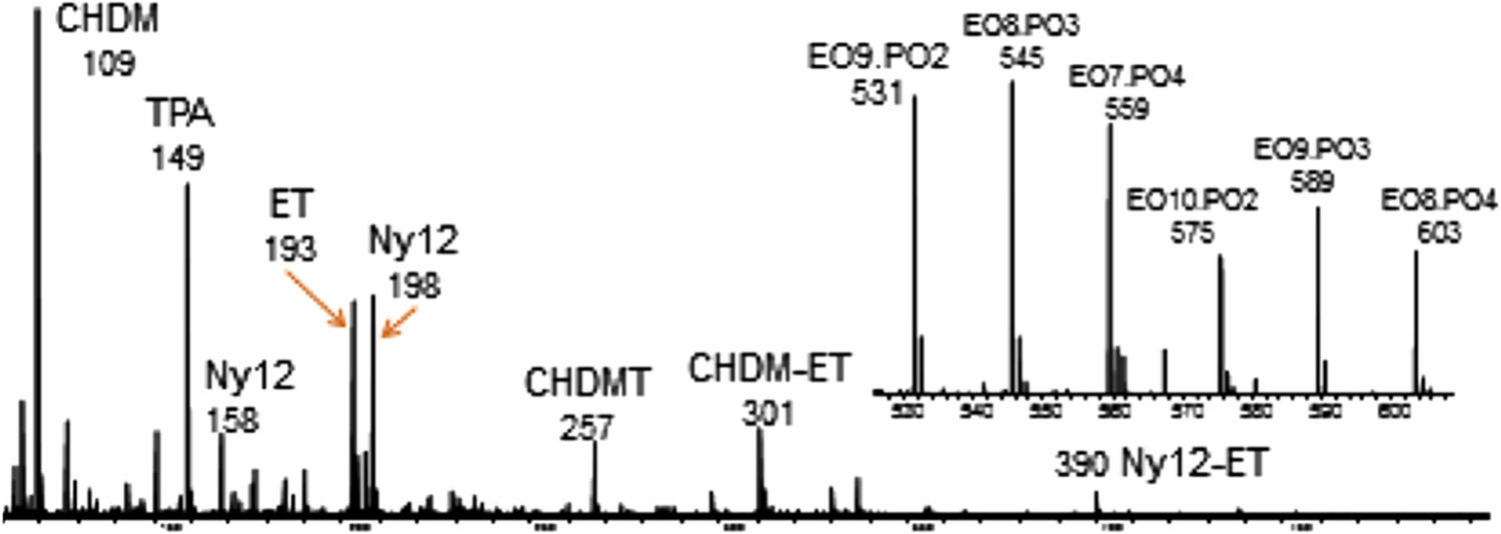
DP‐APCI‐MS of a complex polymer used in an antistatic material, found to contain four different polymers: poly(ethylene terephthalate) (PET), poly(cyclohexanedimethanol terephthalate) (PCHDMT), poly(dodelactam) (Nylon 12) and poly(ethylene oxide‐co‐propylene oxide) (EO‐PO). Adapted with permission from Lattimer and Polce (2011). Copyright 2011 Elsevier.

While ASAP relies on the heated gas stream to induce thermal degradation of polymers, an alternative approach uses a heated direct probe to introduce the sample into the DP‐APCI source to provide finer control of thermal desorption and pyrolysis temperature (Whitson et al., 2008). The authors emphasized that “slow heating allows for temporal separation of the thermal degradation products according to the stabilities of the bonds being cleaved.” In this first publication, a small piece of solid amphiphilic co‐networks was attached to a probe positioned ~2 cm below the corona discharge needle and its temperature was gradually ramped from 100°C to 700°C at a rate of 30°C/min. MS(/MS) analysis of so‐released products provided detailed information about the composition and thermal stability of the investigated polymers. For example, samples with a polyurethane component were observed to release small PDMS oligomers at lower temperature than graft samples. A follow‐up paper described the characterization of commercially available polyurethane samples by DP‐APCI (Whitson et al., 2010). Samples included three polyurethane‐coated polyester fabrics and two elastomeric films. All five samples could be distinguished by their compositions including additives, chain extenders, and polyols. PET was also detected in the fabric samples. DP‐APCI was then reported as a useful technique for the deformulation of insoluble complex samples such as plastic sheets used as antistatic materials (Figure 9), acrylic latex, or paper coated with an acrylic binder (Lattimer & Polce, 2011).

Finally, as for DART and ASAP, DP‐APCI was also valued for the possibility to perform fast screening of additives that usually give strong signals at lower temperature than required for polymer pyrolysis. A screening method for phthalate plasticizers was developed using DP‐APCI coupled with a quadrupole ion trap (Krieger & Schmitz, 2014). In a concurrent study, a variant of DP‐APCI, namely direct‐probe atmospheric pressure photoionization (DP‐APPI) was shown to offer improved selectivity for the targeted brominated flame retardants and plasticizers, and was applied to nontargeted analysis of electronic waste, car interiors, and consumer products (Ballesteros‐Gómez et al., 2014). Although APPI has shown benefits for ionization of small synthetic polymers of low polarity including PIB (Keki et al., 2008), PE (Kéki, Nagy, et al., 2008), and PS (Desmazières et al., 2014; Kuckling et al., 2017), its direct probe version has never been implemented for structural analysis of insoluble polymers.

Overall, techniques based on controlled thermal degradation of insoluble polymers to make them amenable to mass spectrometry have been applied to a large variety of species. This was discussed in this section with a limited number of selected examples, which can be usefully completed by data from Table 3 summarizing reported literature as a function of the methods and the applicable polymer types.

**Table 3:** Controlled thermal degradation techniques used as a function of polymer types.

	DART	TDPy‐DART	ASAP	DP‐APCI
ABS	Black et al. (2017); Cody et al. (2020)	Cody et al. (2020)	Alawani et al. (2023)	
PA	JEOL USA, Inc (2005); Abe et al. (2020); Black et al. (2017); Cody et al. (2020); Forbes et al. (2023); Zhang et al. (2020); Zughaibi & Steiner (2020)	Cody et al. (2020); Liang et al. (2020)	Trimpin et al. (2009); Vitali et al. (2023)	Lattimer and Polce (2011)
PAN	Cody et al. (2020)	Cody et al. (2020); Liang et al. (2020)	Vitali et al. (2023)	
PAO			Siqueira et al. (2018)	
PB	Gaiffe et al. (2019)			
PBS			Barrère et al. (2014)	
PBT		Liang et al. (2020)	Vitali et al. (2023)	
PC			Vitali et al. (2023)	
PDMS	Bridoux and Machuron‐Mandard (2013); Cody and Fouquet (2018); Cody et al. (2020); Gross (2013)	Ashton et al. (2017); Ashton et al. (2021); Cody et al. (2020)	Fouquet, Barrère‐Mangote, et al. (2015)	Whitson et al. (2008)
PE	Black et al. (2017); Cody et al. (2020); Zhang et al. (2020)	Ashton et al. (2021); Cody et al. (2020); Liang et al. (2020)	Barrère et al. (2014); Eissa et al. (2013); Farenc et al. (2017); Vitali et al. (2023)	
PEEK			Cossoul et al. (2015)	
PEO^a^	AlShehri et al. (2022); Bridoux and Machuron‐Mandard (2013); Bridoux et al. (2016); Cody &and Dane (2016); Cody and Fouquet (2018); Cody et al. (2020)	Cody et al. (2020); Yamane et al. (2021)	Beaumesnil et al. (2020); Eissa et al. (2013); Endres et al. (2021); McEwen et al. (2005); Smith et al. (2012); Snyder an Wesdemiotis (2021)	Lattimer and Polce (2011)
PET	Black et al. (2017); Cody et al. (2020); Forbes et al. (2023); Zhang et al. (2020)	Cody et al. (2020); Liang et al. (2020)	Vitali et al. (2023)	Lattimer and Polce (2011); Whitson et al. (2010)
PFPE	Bridoux and Machuron‐Mandard (2013); Cody and Dane (2016)			
PI	Cody et al. (2020)	Cody et al. (2020)		
PIB	Cody and Fouquet (2018); Gaiffe et al. (2019); Nagy et al. (2015)		Beaumesnil et al. (2020); Snyder and Wesdemiotis (2021)	
PLA	AlShehri et al. (2022); Black et al. (2017); Cody and Fouquet (2018); Cody et al. (2020)	Cody et al. (2020)	Barrère et al. (2014)	
PMMA	AlShehri et al. (2022); Bridoux and Machuron‐Mandard (2013); Cody and Fouquet (2018); Cody et al. (2020); Forbes et al. (2023)	Cody et al. (2020)	Barrère et al. (2014); Snyder and Wesdemiotis (2021); Vitali et al. (2023)	Whitson et al. (2008)
PP	Cody et al. (2020); Forbes et al. (2023); Zhang et al. (2020)	Cody et al. (2020)	Barrère et al. (2012); Farenc et al. (2017); Vitali et al. (2023)	
PPI	Bridoux and Machuron‐Mandard (2013)			
PS	JEOL USA, Inc (2005); AlShehri et al. (2022); Forbes et al. (2023); Gaiffe et al. (2019); Zhang et al. (2020)	Ashton et al. (2021); Sato, Nakamura, Takei, et al. (2020)	Smith et al. (2012); Vitali et al. (2023)	
PUR			Alawani et al. (2023); Lebeau and Ferry (2015)	Whitson et al. (2008, 2010)
PVC	AlShehri et al. (2022); Cody et al. (2020)	Cody et al. (2020)		
PVDF	Cody et al. (2020); Pacholski et al. (2023)	Cody et al. (2020)	Gaiffe et al. (2018)	

^a^ also includes other types of polyethers

## 3 CHEMICAL DEGRADATION

Complete or partial degradation of polymers using chemical agents, as increasingly developed for recycling purposes (Coates & Getzler, 2020; Lefay & Guillaneuf, 2023), can also be part of MS strategies for insoluble polymer analysis. Yet, for the macromolecule microstructure to be reconstructed based on chemolysis products characterized by MS, partial and (most importantly) controlled degradation is preferred. The field was pioneered by Montaudo and co‐workers in the early 1990s to produce relatively low molecular weight species amenable to FAB and determine sequential ordering of comonomer units along the chains (Montaudo, 1991), with the help of statistical models (Montaudo & Montaudo, 1992; Montaudo et al., 1991). At that time, the main purpose of chain degradation was to tackle limitations of MS instrumentation, that is, limited chain size for production of molecular ions by FAB and low accessible *m/z* range.

Despite the fact that major advances have been achieved for both ionization (with ESI or MALDI) and mass analysis of large species, chemolysis approaches developed by the Montaudo group have inspired most subsequent works involving analytical chemolysis of polymers and are hence summarized hereafter. Hydrolysis was first proposed for partial degradation of polyamides (Montaudo et al., 1989a) but methanolysis was preferred to best control the size of degradation products. This was demonstrated with copolyesters of microbial origins (Abate et al., 1993; Ballistreri et al., 1989a, 1989b; Ballistreri et al., 1990) as well as for large chains comprised of up to four different co‐monomers (Montaudo et al., 1998). Efficient ammonolysis of aromatic copolycarbonate allowed small bisphenol A blocks to be evidenced in condensation copolymers although these were expected to exhibit an alternate sequence of this monomer with resorcine (Montaudo et al., 1989). When performed with an excess of piperidine at higher temperature, this reaction could also be usefully implemented for copolyesters such as PET (Montaudo et al., 1989b). Preferential methoxidation observed to occur at ether bonds when reacting sodium methoxide with copolymers composed of ethersulfone and ether ketone permitted to identify 44% of random sequence as a result of transetherification that occurs during the reaction expected to produce exactly alternating copolymers (Montaudo et al., 1995). Finally, ozonolysis was found suitable to produce low‐mass species from diene polymers such as poly(isoprene) (PI) and poly(chloroprene) (Montaudo et al., 1992) or butadiene/styrene copolymers (Montaudo et al., 1991).

More recent works involving controlled chemolysis of chains have mostly been reported for accurate determination of the structure and sequence of comonomer units in insoluble copolymers. These degradation processes aim to induce selective backbone bond cleavage by employing chemical agents able to react with specific moieties, which obviously implies prior knowledge of the chemical structure and/or architecture of polymer samples.

**Scheme 1:**
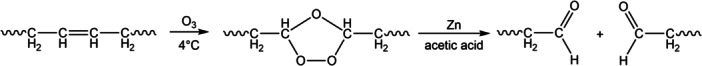
General mechanism for ozonolysis of butadiene‐based (co)polymers. Adapted with permission from Zoller and Johnston (2000). Copyright 2000 American Chemical Society.

For random copolymers, it is essential to keep the degradation reaction sub‐quantitative so that chemolysis products are large enough to derive reliable information regarding composition and distribution of co‐monomers in original chains. For example, the ozonolysis reaction implemented to cleave different butadiene‐based copolymers was controlled for specific exposition times as a function of the size and butadiene content of original chains (Zoller & Johnston, 2000). Doing so, copolymers with molecular weight above 100 kDa could be degraded into chains with mass below 2000 Da as evidenced by MALDI‐MS. The reaction first consists of addition of an ozone molecule across the double bond of butadiene monomers, yielding the ozonide intermediate shown in Scheme 1. To avoid the variety of end‐groups expected upon splitting of this ozonolysis product, reduction with zinc and acetic acid was performed to produce oligomers with aldehyde end‐groups. Ozonolysis was implemented in toluene for soluble substrates and allowed styrene‐butadiene block copolymers to be readily distinguished from their random counterparts: the former yielded polybutadiene homopolymers whereas the latter were observed to degrade into co‐oligomers, which composition was found to closely match (within 5 wt%) expected values. This paper reports similar performance when ozonolysis was performed in the solid state for insoluble acrylonitrile‐butadiene.

**Figure 10:**
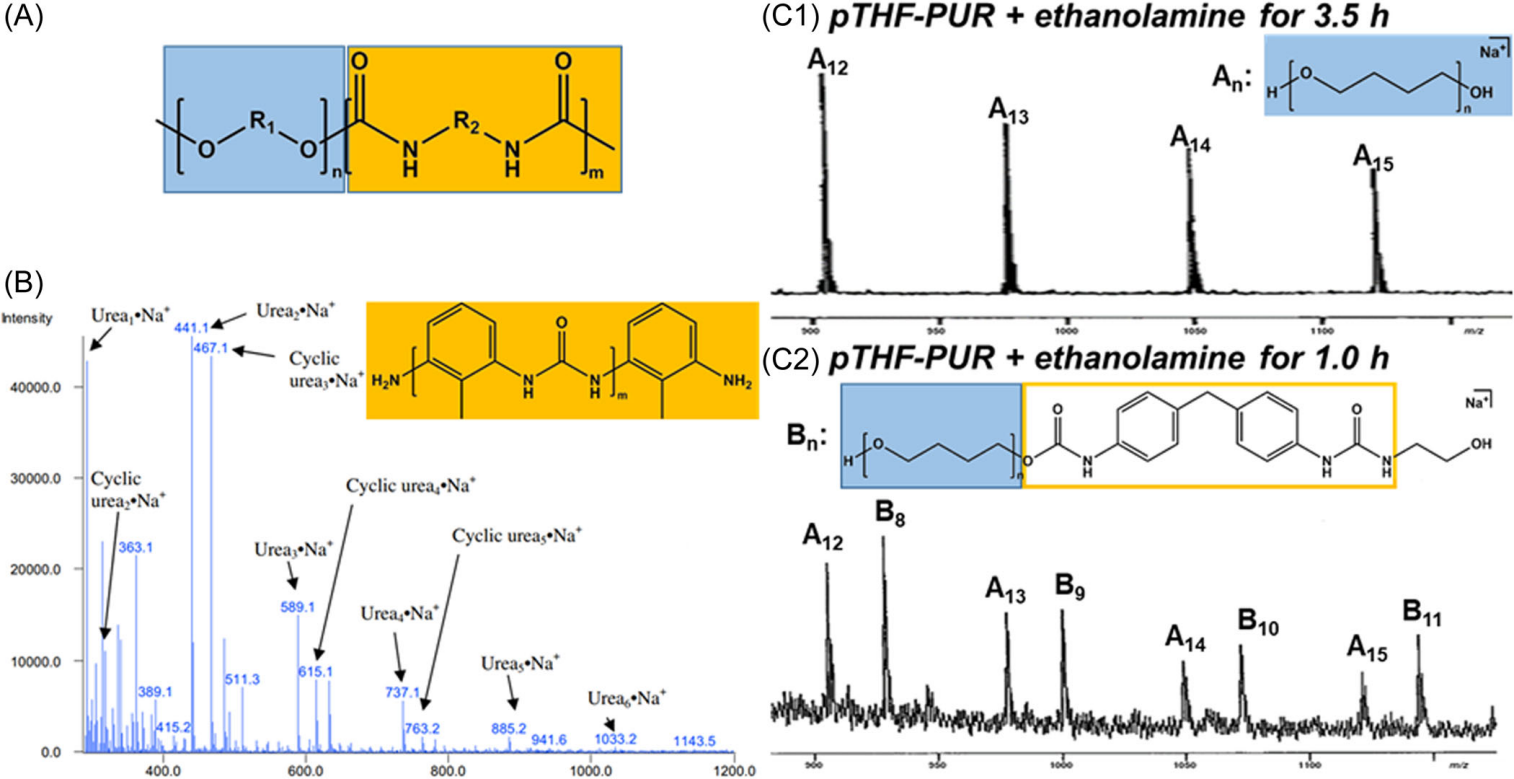
(A) Structure of polyurethanes (PURs) with soft segments (in blue) connected to hard segments (in orange) via a urethane linkage. (B) Matrix‐assisted laser desorption/ionization (MALDI) mass spectrum recorded for hard segments released upon alkaline hydrolysis of toluene diisocyanate (TDI)‐polyether‐foam. Adapted with permission from Aou et al. (2013). Copyright 2013 Elsevier. MALDI mass spectra of soft segments recovered after ethanolamine degradation of PTHF‐PUR copolymer for (c1) 3.5 h or (c2) 1.0 h. Adapted with permission from Mehl et al. (2000). Copyright 2000 American Chemical Society. [Color figure can be viewed at wileyonlinelibrary.com]

In contrast to chemolysis of random copolymers, both selectivity and efficiency are targeted for chemical reactions developed for scission of block copolymers into their constitutive segments. PUR elastomers are the archetypal case of multiblock copolymers that still escape direct MS analysis. Their microstructure combines hard segments alternating with soft blocks, which size and chemical composition define unique macroscopic properties. While small linear PURs can be analyzed as intact gas‐phase species produced by either ESI or MALDI (Crescentini et al., 2019), insolubility of large and/or cross‐linked PURs as found in flexible foams requires selective degradation processes to release either the hard or the soft blocks (Figure 10A) before MS analysis of their length distribution. One reaction implemented to recover intact hard segments of PURs is alkaline hydrolysis, which enables cleavage of ester, urethane, and urea bonds (Mertes et al., 1998). This reaction permits to detach soft blocks from hard segments, the latter being detected with amino end‐groups in MALDI mass spectra. Typically, samples are stirred for 24 h in a water/1‐butanol 50/50 (v/v) solution of NaOH heated to 100°C; then, addition of methanol and subsequent centrifugation of the liquid medium enables hard segments to be recovered as a precipitate. The same protocol was later implemented to characterize by MALDI‐MS the size of hard segments released from poly(urea‐urethane)s (Yontz & Hsu, 2000). Using acid neutralization rather than precipitation in methanol was shown to improve recovery of the shortest hard segments (Aou et al., 2013), as exemplified with MALDI‐MS data shown in Figure 10B. Acid‐catalyzed hydrolysis has also been proposed to selectively degrade soft polyester blocks and liberate intact hard segments with hydroxyl end‐groups evidenced by MALDI‐MS, as reported for PURs prepared with poly(butylene adipate) (PBA) polyols (Murgasova et al., 2002).

Alternative strategies targeting recovery of soft segments were first reported by Hercules and coworkers (Mehl et al., 2000). An excess of ethanolamine (at 150°C for 3.5 h under N_2_ atmosphere) was employed to cleave urethane bonds in polyether‐based PURs such as those comprised of poly(tetrahydrofuran) (PTHF) soft blocks. After vacuum removal of ethanolamine, the residue was washed with ether and subjected to MALDI, yielding a distribution of hydroxyl‐terminated PTHF oligomers (Figure 10,c1). Interestingly, reducing the reaction time enabled detection of a second distribution of PTHF oligomers still having the PUR diisocyanate linkage (Figure 10,c2), hence allowing information on both the average size of PTHF segments and the diisocyanate used for PUR synthesis. Since ethanolamine also cleaves ester bonds, the more selective phenylisocyanate reagent had to be employed to recover intact soft blocks from polyester‐based PUR (Mehl et al., 2000). When implemented for PBA‐based PURs, MALDI mass data show that the reaction mostly produced PBA oligomers with the expected N‐phenylurethane terminations together with minor series of ions enabling identification of the diisocyanate linkage. Using a series of model PURs prepared from 4,4′‐diphenylmethane diisocyanate (MDI) and a mixture of PBA diol‐terminated polyesters, Hercules and coworkers further improved this approach with a methodology that combines (i) phenylisocyanatolysis to release soft blocks and (ii) partial acid‐catalyzed hydrolysis of PBA to recover the hard blocks as hydroxyl‐terminated species, using MALDI‐MS to characterize both products (Murgasova et al., 2002).

**Figure 11:**
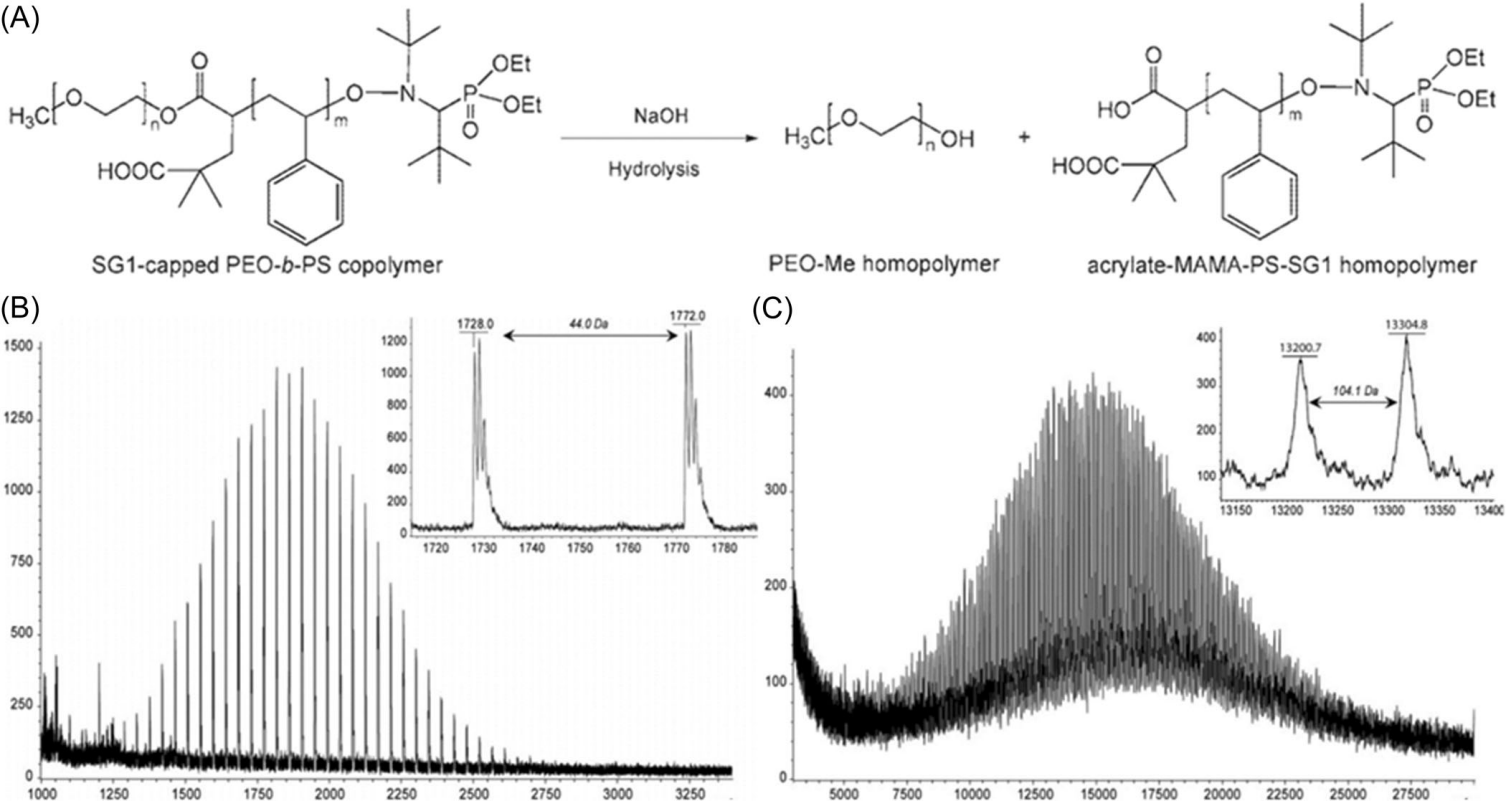
(A) Selective hydrolysis of the ester bond in the junction group of PEO‐*b*‐PS to produce two homopolymers that could be individually characterized by MALDI‐MS as (B) PEO, using DHB/NaI and (C) PS, using DCTB/AgTFA. Adapted with permission from Girod et al. (2009). Copyright 2009 Wiley.

The original capability of amphiphilic copolymers composed of poly(ethylene oxide) (PEO) hydrophilic segments and PS hydrophobic blocks to arrange into micelles is highly relevant in material sciences but becomes a major drawback for their analysis. Selective decrease of local mobility in micellar aggregates affects solution NMR data, lack of appropriate standards makes SEC calibration complicated whereas selection of proper matrix for MALDI can become a tedious task since conditions dictated by one block are most often not compatible with requirements of the second block (Pizzala et al., 2021). Taking advantage of the ester bond in the junction group of PEO‐*b*‐PS prepared by nitroxide‐mediated polymerization (Figure 11A), Girod et al. optimized an hydrolysis reaction to cleave these macromolecules into their constituting blocks (Girod et al., 2009). Under basic conditions (NaOH in THF/water 90/10, v/v), specific hydrolysis of the targeted ester bond was demonstrated for a series of copolymers with various block sizes. After 72 h at 70°C, the reaction medium was evaporated to dryness, dissolved in a 50/50 (v/v) dichloromethane/water mixture and polymers recovered from the organic phase in which PEO could be separated from PS by selective precipitation. Each homopolymer could thus be individually mass analyzed with established MALDI methods, using 2,5‐DHB/NaI for PEO (Figure 11B) and DCTB/AgTFA for PS (Figure 11C).

**Scheme 2:**
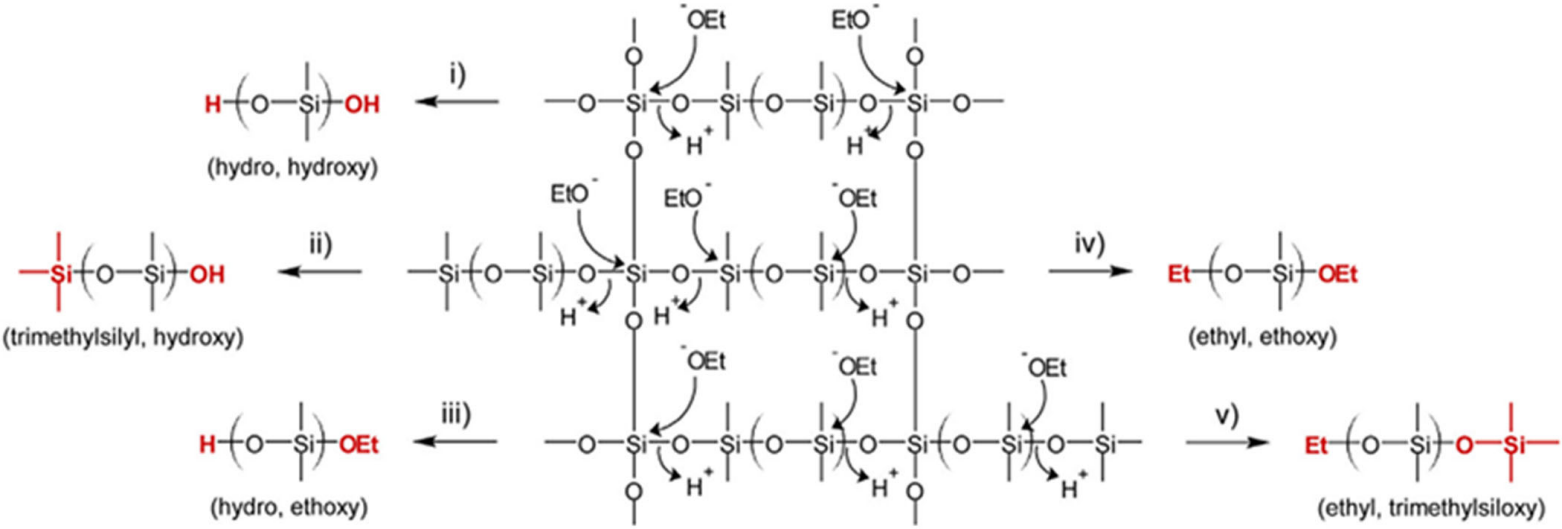
Ethanolysis for controlled deconstruction of the network found in silicon‐based plasma‐polymers. Reprinted with permission from Fouquet (2015). Copyright 2015 Elsevier. [Color figure can be viewed at wileyonlinelibrary.com]

Besides copolymers, controlled chemolysis also proved relevant to get unique insights in the architecture of networks. In an approach named “network disassembly spectrometry,” a site‐selective reaction was developed to quantify the fraction of primary loops and the numbers and structures of dangling chains in PEG hydrogels (Zhou et al., 2012). Hydrolysis of ester bonds under basic conditions permitted to break the network into specific products that could be unambiguously assigned in liquid chromatography (LC)‐ESI‐MS due to their unique mass and further quantified in LC‐UV. Another chemolysis approach was developed for the controlled deconstruction of plasma polymers obtained as reticulated thin films after passage of gaseous monomers through a plasma discharge (Biederman, 2004). Due to their particular network organization, plasma polymers display unique surface properties but also major analytical pitfalls related to their very low solubility in common solvents. For coatings formed by plasma polymerization of silicon‐based monomers, Fouquet and coworkers conceived a chemolysis strategy where nucleophilic attack of ethoxylate anions on silicon atoms linked to three to four oxygen atoms (as found for cross‐linking and branching points, respectively) allowed the constituting pieces of the network to be released as small oligomers readily ionized by ESI (Fouquet, 2014). As first established for oligomer models (Fouquet et al., 2012), the degradation process was sufficiently controlled to safely deduce, from MS/MS end‐group analysis of ethanolysis products, the original architecture of the network. For solid plasma polymers, thin films were collected from the electrodes, finely ground, and swelled in tetrahydrofuran overnight to eliminate residual monomers. The solid sample was then submitted to degradation using an ethanolic solution of KOH (50 mg/mL) at room temperature for complete dissolution after 6 h. Aliquots of the reaction medium were diluted in a methanolic solution of ammonium acetate to favor ESI production of ammonium adducts for best MS/MS end‐group analysis (Fouquet et al., 2013, 2011). Reconstruction of the pristine architecture is indeed based on the fact that terminations of oligomers detached from the network reveal their original location (Fouquet et al., 2013). This is illustrated in Scheme 2, with segments between two cross‐linking points released as oligomers ended by H/OH (pathway i), H/OEt (pathway iii), and Et/OEt (pathway iv) whereas species detected with only one termination diagnostic of the ethanolysis process (either HO in pathway ii or Et in pathway v) can be assigned to small chains pending from the network. Oligomers with none of these end‐groups were also detected, revealing that free plasma‐polymerized chains can be embedded in the final network. Dimension of meshes as well as reticulation degree of the network were then derived from average molecular masses calculated for the different oligomeric series. Data obtained for plasma‐polymers created from hexamethyldisiloxane (Fouquet, 2015) and octamethylcyclotetrasiloxane (Fouquet et al., 2013) were consistent with information obtained by quantitative ^29^Si solid state NMR.

## 4 REACTIVE IONIZATION

A new emergent strategy to make insoluble synthetic polymers amenable to mass spectrometry is to implement a reactive mode of ionization, which is achieved when one additive used for sample preparation reacts with the analyte during the ionization process. Formation of such derivatives is often considered as an undesired reaction but can also be advantageously used for analytical purposes. For example, covalent attachment of matrix molecules with aromatic ketone or aldehyde functionalities to synthetic polymers containing primary amine groups was reported as a useful reactive MALDI method for precise determination of the number of NH_2_ end‐groups (Zaikin et al., 2015). As compared to sample pre‐treatments reviewed in the previous section, implementing reactive ionization to induce chemolysis of insoluble synthetic polymers offers major advantages of speed and lack of sample preparation, as highlighted hereafter for two recently reported methodologies.

### 4.1 Reactive surface‐assisted laser desorption/ionization (reactive SALDI)

**Figure 12:**
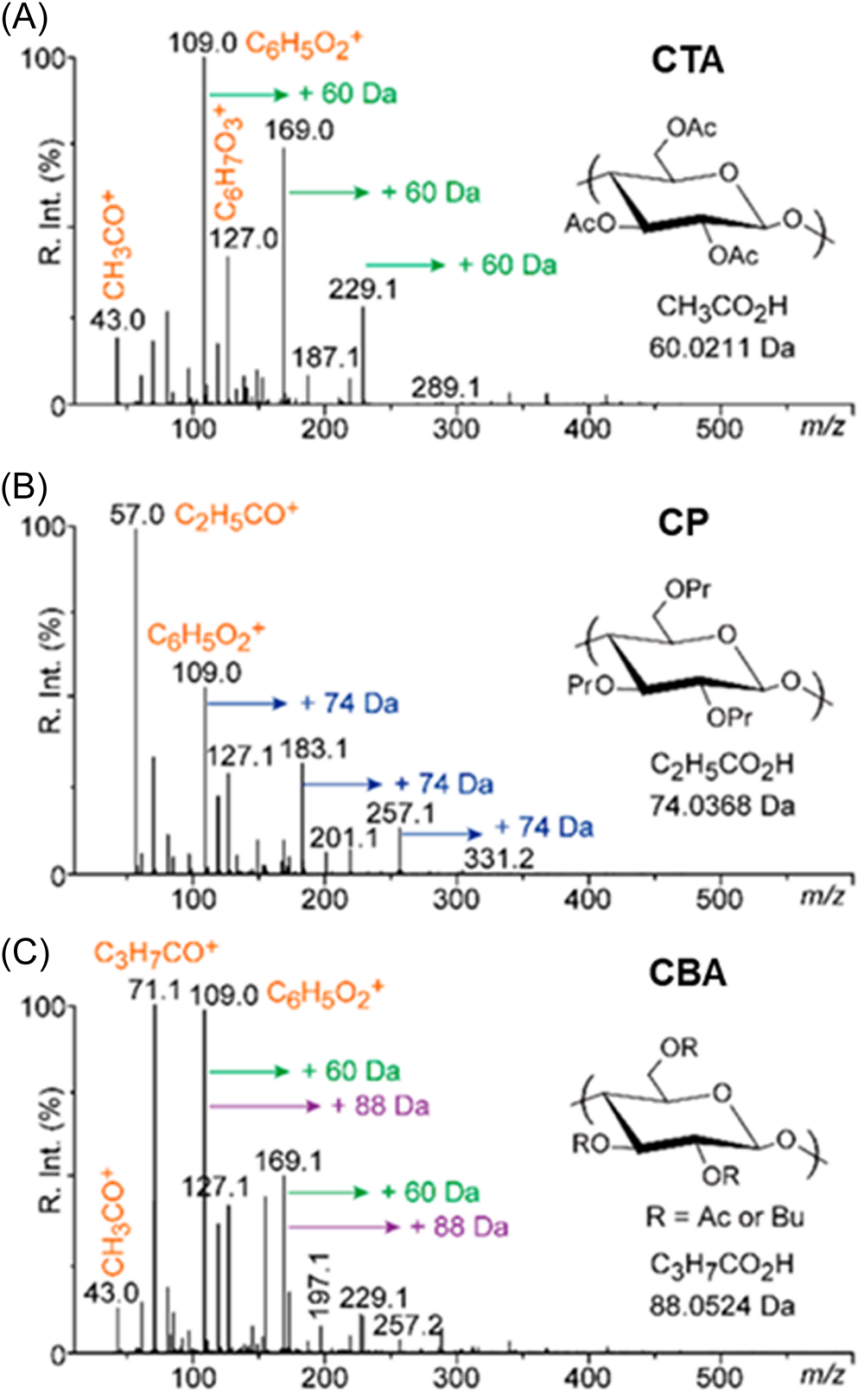
Reactive surface‐assisted laser desorption/ionization mass spectra recorded from DIUTHAME chip for cellulose samples functionalized with (A) acetate, (B) propionate, and (C) acetate and butyrate groups. Adapted with permission from Fouquet, Cody, et al. (2020). Copyright 2020 American Chemical Society. [Color figure can be viewed at wileyonlinelibrary.com]

In contrast to MALDI employing a matrix, SALDI takes advantage of the absorption capabilities of nanostructured substrates to promote desorption/ionization of analytes (Sunner et al., 1995). When implemented on through‐hole alumina membrane (so‐called DIUTHAME technique) (Naito et al., 2018), a reactive mode of SALDI was discovered to induce cleavage of different types of bonds in synthetic polymers (Sato, Nakamura, Fouquet, et al., 2020). This reaction is actually catalyzed by traces of phosphoric acid from the elaboration of DIUTHAME chip and was usefully employed to qualify a variety of high molecular weight synthetic polymers from their reactive SALDI‐MS fingerprint (Fouquet, Cody, et al., 2020). For example, protonated monomers were the main signature for poly(caprolactone) (PCL), PET, monadic nylons 6 and 12 as well as dyadic nylons 6/6 and 6/12. As documented in Figure 12, detailed structural features could be obtained for functionalized celluloses. Mass spectra recorded upon reactive SALDI of the three samples investigated in this study share the same *m/z* 109.0 signal, assigned to fully dehydrated glucose. From this signature ion, distinct additional patterns are observed as a function of the chemical moieties functionalizing cellulose: acetate (Figure 12A) and propionate (Figure 12B) are respectively revealed by +60 and +74 Da mass shifts whereas ions detected at +60 or +88 Da (or different combinations thereof) indicate that the third cellulose sample is functionalized with both acetate and butyrate moieties (Figure 12C).

When applied to random copolymers of lactic acid (LA) and glycolic acid (GA), quantitative performance of the method was also demonstrated with accurate determination of their LA/GA content (Fouquet, Pizzala, et al., 2020). Reactive SALDI was observed to induce partial cleavage of C–O bonds in the skeleton of these macromolecules, yielding series of small co‐oligomeric ions of the form C_3_H_3_O‐(C_3_H_4_O_2_)_*x*_(C_2_H_2_O_2_)_*y*_^+^, with *x* and *y* indicating the number of LA and GA units, respectively. In good agreement with ^1^H NMR data, the LA content computed from reactive SALDI mass data was 75% for PLAGA 75/25, 65% for PLAGA 65/35, and 40% for PLAGA 50/50. With similar results obtained for the whole polymers or SEC fractions thereof, efficiency of the chemolysis process was shown to be independent of chain length.

### 4.2 Reactive desorption electrospray ionization (reactive DESI)

**Figure 13:**
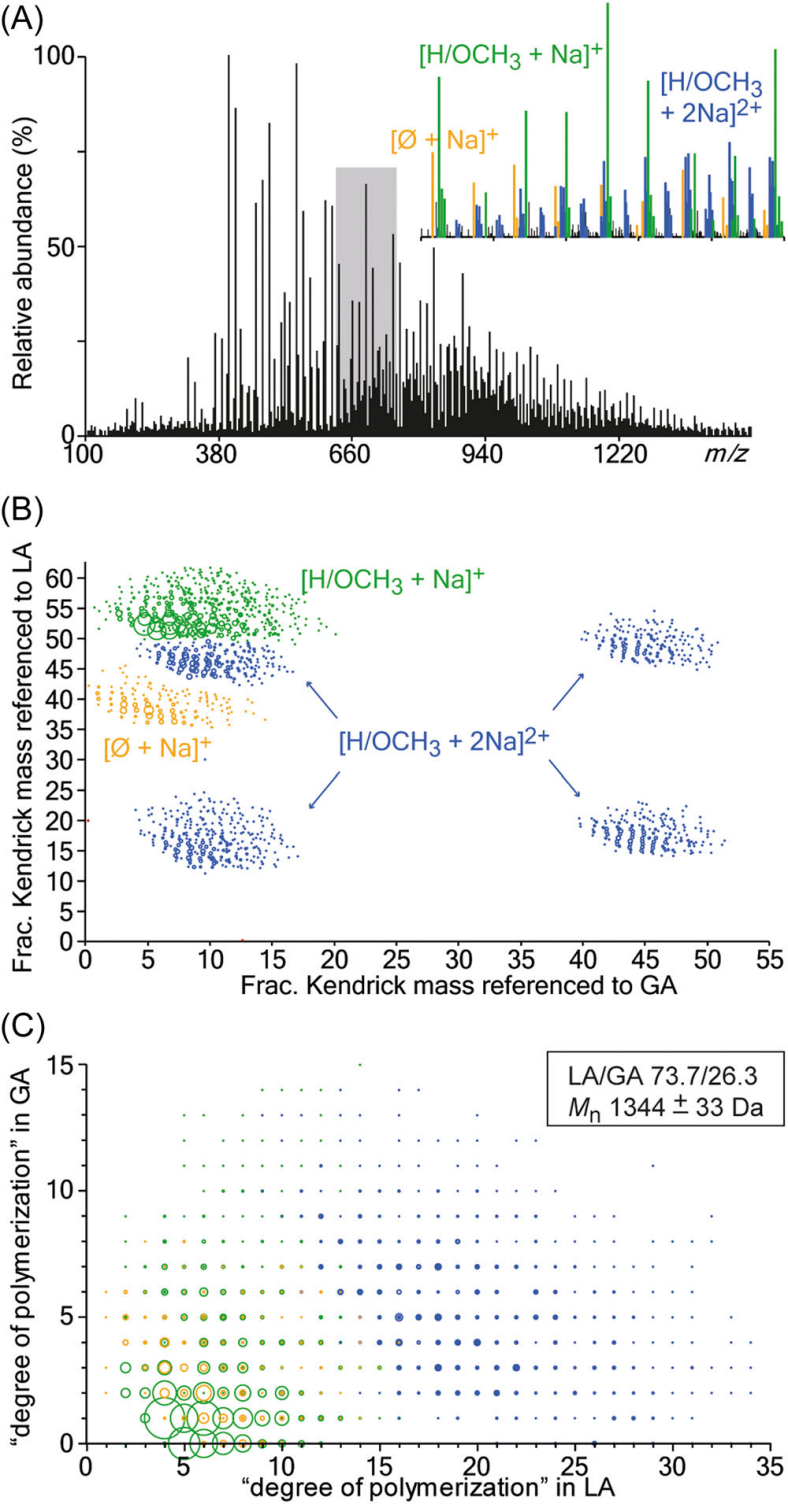
(A) Mass spectrum of PLAGA 65/35 recorded upon reactive desorption electrospray ionization with a methanolic solution of NaOH. (B) “Referenced” Kendrick plot, enabling discrimination of ion series based on their end groups and charge states and further used to obtain (C) degree of polymerization (DP) plot. Adapted with permission from (Fouquet et al., 2021). Copyright 2021 American Chemical Society. [Color figure can be viewed at wileyonlinelibrary.com]

In‐source chemolysis of insoluble copolymers was also achieved with DESI, an ambient ionization method that makes use of high‐velocity electrosprayed droplets to sample analytes from surfaces (Takáts et al., 2004). Performing DESI in a reactive mode consists of adding reagents in the sprayed solution to induce specific ion/molecule reaction at the sample surface. For example, a reactive DESI method employing dithiothreitol was developed to cleave disulfide bonds linking small oligomers to polyacrylamide hydrogel and hence release these coded taggants for on‐line MS/MS sequencing (Youssef et al., 2022). Recently, solvolysis of insoluble PLAGA substrates was successfully achieved by reactive DESI and allowed the erosion susceptibility of these degradable copolymers to be evaluated from the LA/GA composition of released co‐oligomers (Fouquet et al., 2021). The method takes advantage of the accelerated rate of reactions occurring in the unique environment of charged droplets (Badu‐Tawiah et al., 2012) to induce efficient methanolysis of PLAGA chains. Although hydrolysis is traditionally used as a degradation process for these polyesters, water was shown to raise issues on the ionization side with properties such as high surface tension being incompatible with optimal formation of small droplets. Instead, methanol was employed to best fulfill the dual role of ESI solvent and chemolysis reagent. Methanolysis was performed in alkaline conditions, using NaOH to also introduce sodium as the cationizing agent. These reactive DESI experiments produce PLAGA co‐oligomers with H/OCH_3_ terminations, as expected upon methanolysis of ester bonds, and of sufficient large size for some species to be detected at the +2 charge state (Figure 13A). Yet, inventory of methanolysis products was best achieved after these complex mass data have been processed by Kendrick analysis (Figure 13B), further using such 2D maps to conveniently extract quantitative information such as average co‐monomer composition and *M*_*n*_ value (Figure 13C). Minimal preparation was requested for samples to be exposed to the DESI probe: soluble polymers were simply film‐casted whereas the poorly soluble GA‐rich substrates were prepared as solid pellets molded with a hydraulic press. Mass spectra of similar quality were recorded for all investigated samples, with LA/GA ratios ranging from 100/0 to 5/95 and average molecular weight between 10 and 180 kDa. Computing reactive DESI mass data for this set of samples showed that the extent of methanolysis increases linearly (*R* ^2^ = 0.9900) with the GA content in original substrates, consistent with the susceptibility of ester bonds toward solvolysis being higher in GA than in LA units.

## 5 CONCLUSIONS AND PERSPECTIVES

After ESI and MALDI have been used in a nearly exclusive manner for years to perform MS of synthetic polymers, challenges raised by insoluble samples of either very high molecular weight or reticulated architectures have renewed the interest for analytical degradations performed in old MS strategies. Controlled degradation processes are implemented either in the ionization source on‐line with MS or in an off‐line manner to produce samples whose components are amenable to ESI or MALDI. In both cases, these approaches permit to generate gas‐phase ions that are diagnostic of polymeric chains that would otherwise escape MS analysis. Once formed, these ionic species can be subjected to any experiments offered by most modern instrumentation (MS, MS/MS, IMS, …). Sources operating at relatively low temperatures (≤500°C) such as DART, ASAP, and DP‐APCI yield oligomeric species that retain molecular connectivity details, most often revealed by MS/MS experiments. Major benefits of these approaches include rapid analysis, minimal (if any) sample preparation and no need for prior knowledge of the questioned polymeric materials. Variable temperature ramping in both ASAP and DP‐APCI allows early‐desorbing additives to be removed from polymer mass spectra, and data interpretation can be further simplified using specific data treatment such as KMD, high resolution mass analyzer or IM‐MS couplings. As compared to DART, ASAP involves more energetic processes and can induce thermal degradation that can provide complementary information. For example, when investigating food packaging for the migration of polyester oligomers used to increase their mechanical stability, it was found that DART enabled detection of longer chains (up to *m*/*z* 1000) whereas ASAP was best at highlighting monomeric species observed as the lowest *m*/*z* ions (Osorio et al., 2022). In great contrast to thermal degradation, chemical processes to be implemented for deconstruction of long chains or disassembly of networks obviously imply prior knowledge of the polymer samples. Such targeted approaches can be performed off‐line during a specific bulk sample pretreatment or implemented in‐line with MS with the ionization source serving as a reaction chamber. Most importantly, chemolysis needs to be highly controlled to generate degradation products that still contain reliable information. For example, while developing a depolymerization method based on a metathesis reaction to generate end‐derivatized oligomers from polydienes, Rolando and coworkers had to employ 2,6‐dichloro‐1,4‐benzoquinone to prevent olefin isomerization (Mahmoud et al., 2020). As a nice summary for this review, the latter study also employed ASAP‐IM‐MS and DP‐APCI coupled to FT‐ICR MS, as well as pyrolysis comprehensive two‐dimensional gas chromatography mass spectrometry (Py‐GCxGC‐MS), to obtain complementary information on these highly insoluble polymers.

## AUTHOR CONTRIBUTIONS

**Thierry N. J. Fouquet**: Writing—original draft; writing—review and editing. **Robert B. Cody**: Writing—original draft; writing—review and editing. **Laurence Charles**: Conceptualization; writing—original draft; writing—review and editing.

## CONFLICT OF INTEREST STATEMENT

The authors declare no conflict of interest.
